# Novel Worker‐Like Behavior Observed in Gynes of the Social Parasite *Tetramorium microgyna*

**DOI:** 10.1002/ece3.72960

**Published:** 2026-01-22

**Authors:** François Brassard, Christina Kwapich

**Affiliations:** ^1^ Charles Darwin University Darwin Northern Territory Australia; ^2^ School of Agriculture and Environment The University of Western Australia Crawley Western Australia Australia; ^3^ Department of Biology University of Central Florida Orlando Florida USA

**Keywords:** Formicidae, inquiline, social parasitism, South Africa, *Tetramorium*

## Abstract

Socially parasitic ants increase their own fitness by exploiting the labor and resources of non‐kin ant colonies. Here, we report a novel worker‐like behavior observed in an African workerless inquiline, *Tetramorium microgyna* , a parasite of *Tetramorium sericeiventre* and *Tetramorium sepositum* . We observed several *T. microgyna* gynes excavating soil and performing nest maintenance tasks at the entrance of an established *T. sericeiventre* host colony. We photographed this event in nature, then dissected *T. microgyna* gynes to establish mating status and reproductive capacity. All *T. microgyna* gynes that participated in excavation behaviors were unmated, with ~6 ovarioles. We hypothesize nest excavation by inquilines represents an artifact of a non‐parasitic past, where gynes that fail to mate and disperse remain in their natal colony and assume a secondary, but still mutually beneficial worker‐like role. While nest excavation by socially parasitic foundresses could be an artifact of an ancestral behavioral repertoire associated with independent colony founding, this possibility is unlikely, because the behavior occurred in the presence of a robust worker population. While helping behavior in post‐reproductive inquilines does not increase personal fitness, it also does not reduce it, and may be maintained through relaxed selection. Although host and parasite gynes are morphologically distinct, a third possibility is that putative *T. microgyna* parasites are actually microgynes of their ‘host’ species. Socially parasitic ants are rarely found and poorly studied compared to their non‐parasitic counterparts. Our findings provide insights into how selection may act on developmental and behavioral programs during the evolution of social parasites from non‐parasitic ancestors.

## Graphical abstract

Our article reports, to our knowledge, the first observations of inquiline gynes performing worker tasks for their host colony. We dissected these gynes to establish that they were all unmated. We hypothesize that nest excavation by social parasites represents an artifact of a non‐parasitic past.

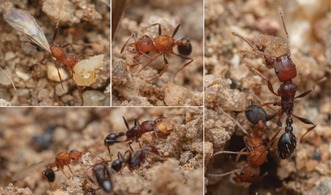

## 1 Introduction

**FIGURE 1:**
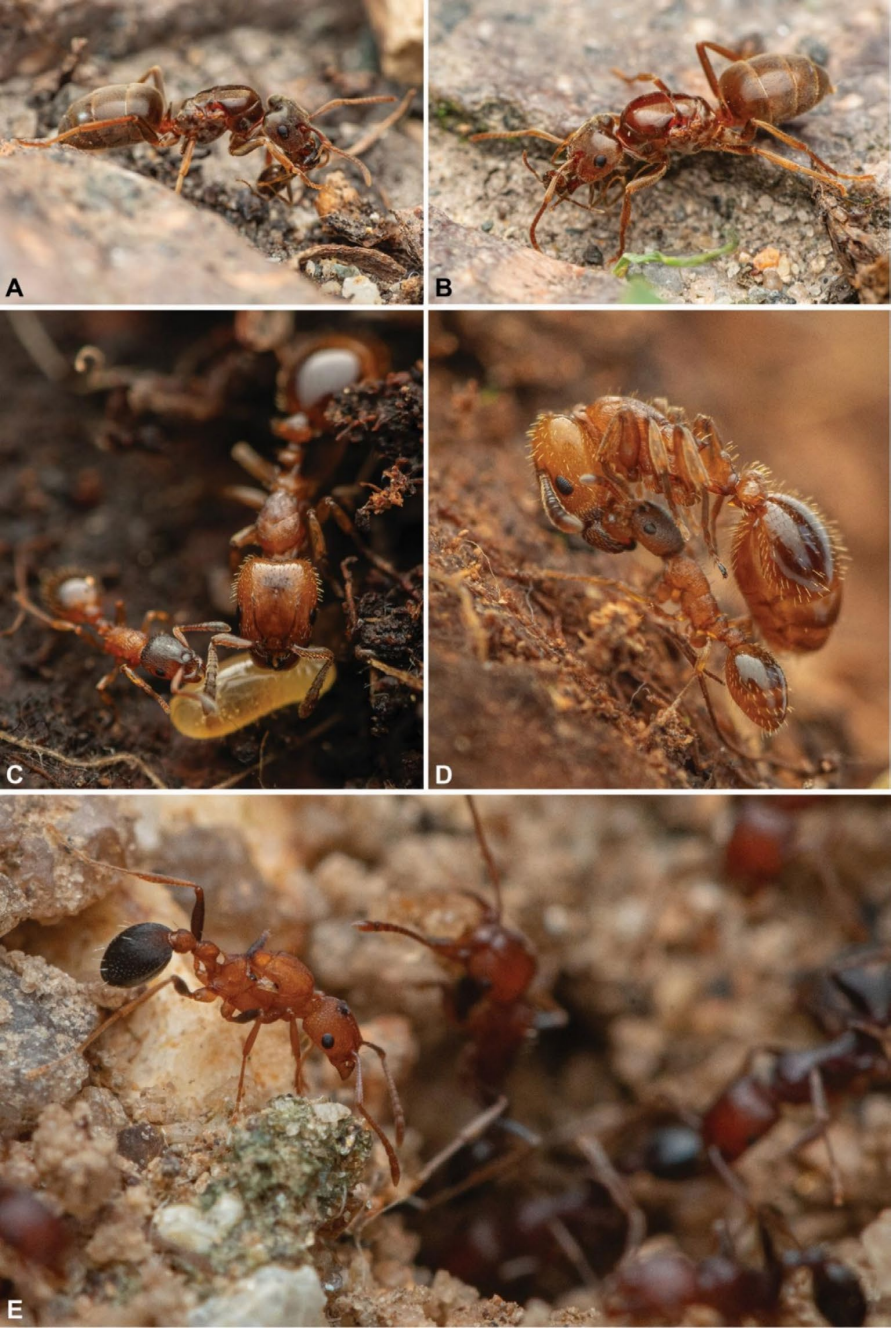
Types of social parasitism are (A, B) temporary social parasitism, (C, D) dulosis and (e) inquilinism. In (A, B) two gynes of temporary social parasite species in the genus *Lasius* captured and chewed workers of a host *Lasius* species, likely to gain their odor before invading the host colony. In (C, D) the host species are either *Leptothorax acervorum* or *Leptothorax muscorum* , whereas the parasite is the European robber ant *Harpagoxenus sublaevis* , which is listed as vulnerable by the IUCN (Social Insects Specialist Group 1996b). In (D), a worker of the *Leptothorax* host species transports a worker of the Harpagoxenus parasite species. In (E), a wingless gyne of the inquiline species *Tetramorium microgyna* is about to enter the nest of its host species *Tetramorium sericeiventre* . Photos by François Brassard.

A key feature of eusocial insect societies is the division of labor between a reproductive caste and a non‐reproductive worker caste responsible for brood care, nest maintenance, foraging and defense. Socially parasitic ants increase their own fitness by exploiting the labor and resources of established, non‐kin ant colonies through temporary social parasitism, dulosis, or inquilinism (Figure 1).

**FIGURE 2:**
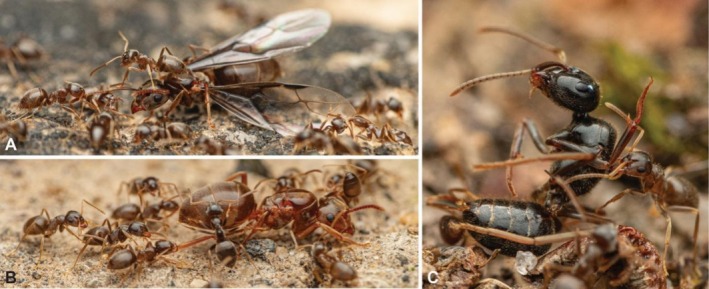
Instances of failed invasion attempts by gynes of temporary social parasites. Here, all parasites and hosts belong to the genus *Lasius*. Photos by François Brassard.

Temporary social parasites exploit their hosts only during the initial stages of colony founding. Typically, a temporary social parasite queen will undertake the risky venture of invading a host nest, sometimes by first killing and interacting with a host worker's corpse, supposedly to obtain colony‐specific recognition odors (Gösswald 1938; Buschinger 2009) (Figures 1A,B and 2). Once inside the nest, the parasite exploits the host colony's workforce to raise her own worker brood, sometimes killing the resident host queen. However, once the parasite's brood mature, parasite workers take care of subsequent generations of parasite workers and thus become independent from their hosts (Buschinger 2009; Rabeling 2021). Dulotic social parasite species also usurp a host species nest by killing the resident queen, but unlike temporary parasites, they are permanently parasitic. Workers of dulotic species usually cannot forage, care for brood, perform nest maintenance tasks, or even feed themselves (Buschinger 2009), but specialize in periodically conducting ‘kidnapping raids’ to capture worker pupae of a host species. After eclosing, these captured host workers take care of the parasitic colony's needs (D'Ettorre and Heinze 2001; Buschinger 2009).

Inquilinism is the most extreme form of social parasitism, as inquiline species typically lack a worker caste and only produce sexuals (i.e., males and queens) (Sumner et al. 2003; Buschinger 2009; Rabeling 2021). Inquiline queens invade an established host nest, but in contrast to temporary social parasites and dulotic species, they usually coexist with the queens of the host species and rely on host queens to produce workers that rear their parasitic, sexual offspring (Buschinger 2009). Most inquilines are substantially smaller than their host queens, and some flank or even attach themselves to their host queen (Buschinger 1986; Davis and Deyrup 2006; Johnson et al. 2008; de la Mora et al. 2020).

**FIGURE 3:**
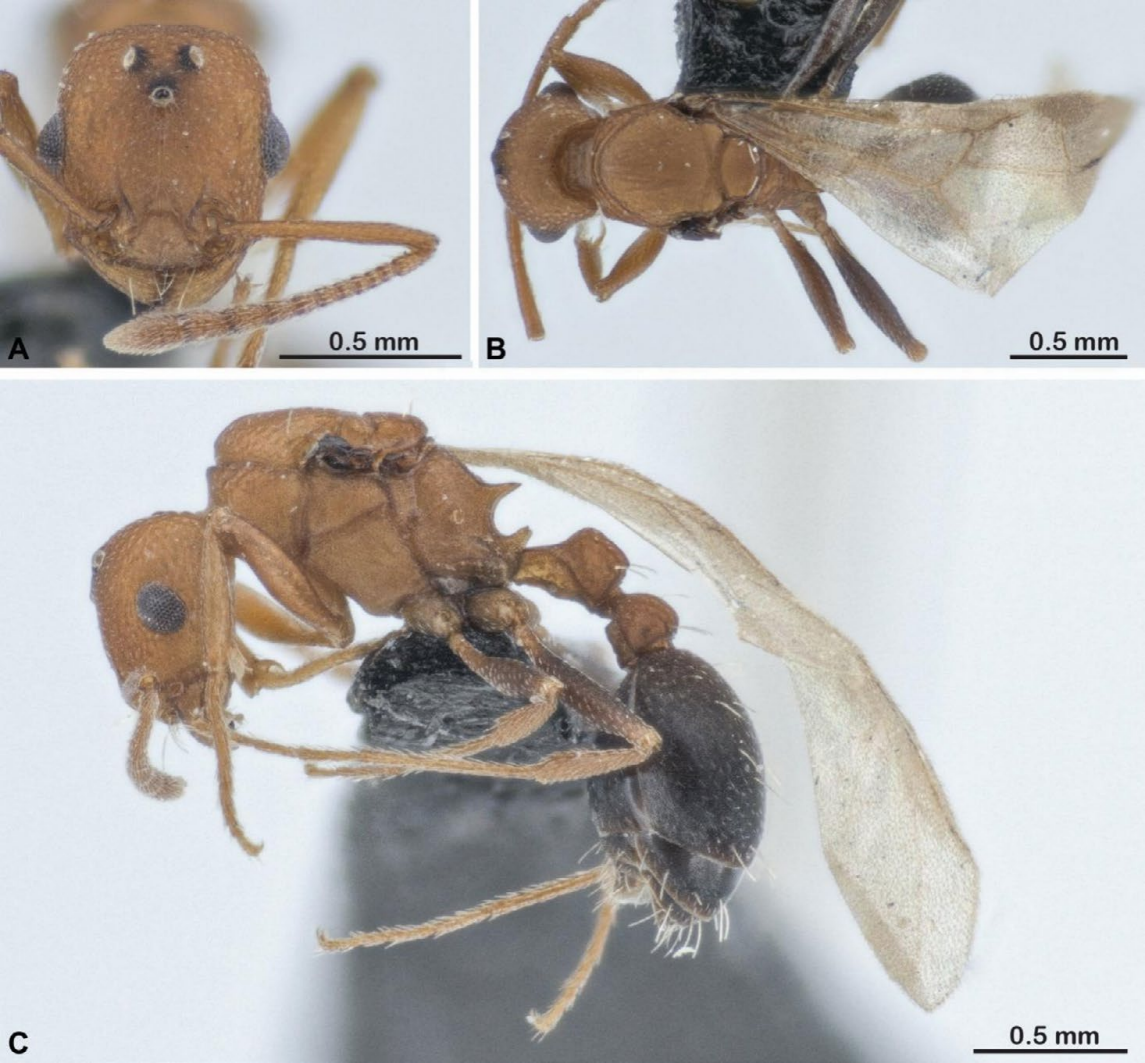
Photos of (A) head view, (B) *dorsal* view and (C) *lateral* view *of a g*yne of *Tetramorium microgyna* . Photos by François Brassard.

**FIGURE 4:**
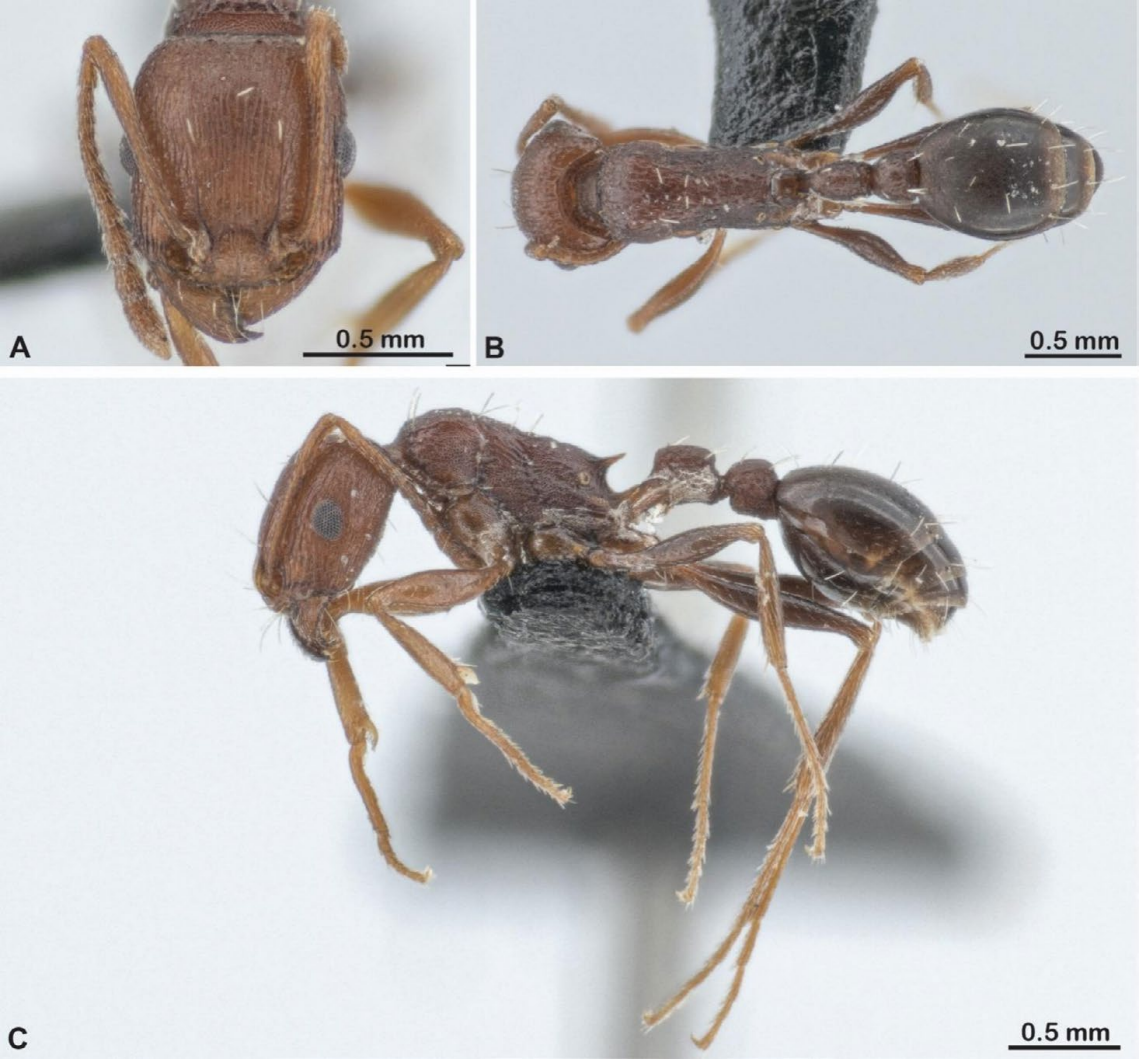
Photos of (A) head view, (B) *dorsal* view and (C) *lateral* view *of a w*orker of *Tetramorium sericeiventre* . Photos by François Brassard.

The inquiline species *Tetramorium microgyna* (Figure 3) is a known parasite of *T. sericeiventre* (Figure 4) and *Tetramorium sepositum* (Bolton 1980). *Tetramorium microgyna* is listed as ‘vulnerable’ on the IUCN (Social Insects Specialist Group 1996a), like most other socially parasitic ants (Mabelis 2007; Alonso 2010). *Tetramorium microgyna* occurs in Angola, South Africa and Zimbabwe, which largely coincides with the distribution of its host *T. sepositum* (South Africa and Zimbabwe), and with the southernmost range of its host *T. sericeiventre* , which occurs throughout most of Africa (Bolton 1980; Janicki et al. 2016; AL‐Keridis et al. 2021; Evan Economo and Benoit Guénard 2024). Besides this information, virtually nothing is known about the natural history of *T. microgyna* .

Here, we report observations of *T. microgyna* gynes performing nest excavation behaviors made during a chance encounter. In non‐parasitic ant lineages, gynes that fail to mate sometimes stay in their natal colony and perform worker‐like tasks (Table 1), but such ‘helper gyne’ behavior is unexpected and novel for a workerless, social parasite. To determine why parasitic gynes might behave like workers in host colonies, we dissected them and established their mating status, reproductive and dispersal potential.

**TABLE 1:** Worker‐like or independent foundress‐like behaviors performed by non‐reproductive gynes (not comprehensive).

Subfamily	Species	Parasitic?	Location	Wings	Behavior	Source
Myrmicinae	*Tetramorium microgyna*	**Yes**	Field	**Alate, partially dealate,** dealate	Excavating soil alongside at nest entrance, *T. sericeiventre* host workers	This study
	*Tetramorium* sp. (*impressum* gp.)	No	Field	Dealate	Excavating soil at nest entrance, alongside workers	This study
	*Acromyrmex echinatior*	No	Lab	Dealate	Brood care, defense	Nehring et al. (2012)
	*Acromyrmex niger*	No	Lab	Dealate	Foraging	Della Lucia et al. (1993)
	*Acromyrmex octospinosus*	No	Lab	Dealate	Brood care, defense	Nehring et al. (2012)
	*Acromyrmex subterraneus*	No	Lab	Dealate	Foraging	Della Lucia et al. (1993)
	*Mycetomoellerius turrifex*	No	Field, Lab	Dealate	Excavation, guarding, carcass tending, brood care, fungus garden care	Murakami (2020)
	*Mycetomoellerius urichii* (formerly *Trachymyrmex fuscus*, see Weber 1958; Solomon et al. 2019)	No	Field	N.R.	Foraging	Araújo et al. (2015)
	*Pogonomyrmex pima*	No	Field	Dealate	Foraging	Johnson et al. (2007)
	*Veromessor andrei*	No	Field	Dealate	Foraging, midden work	Creighton (1953); Brown (1999)
Ponerinae	*Harpegnathos saltator*	No	Lab	Dealate	Worker‐like dominance behaviors	Pyenson et al. (2022)
	*Neoponera apicalis*	No	Lab	**Alate**	Foraging	Fresneau and Dupuy (1988)
	*Odontomachus rixosus*	No	Field, lab	Dealate	Foraging, allogrooming, larval care	Ito et al. (1996)
*Ectatomminae*	*Ectatomma tuberculatum*	No	Lab	N.R.	Patrolling, foraging	Hora et al. (2005)
	*Ectatomma vizottoi*	No	Lab	Dealate	Brood care, grooming, foraging	Vieira et al. (2012)

*Note:* Observations of such behaviors in parasitic species and alate gynes are shown in bold.

Abbreviation: N.R., not reported.

## 2 Material and Methods

We found a nest of *T. sericeiventre* parasitized by the social parasite *T. microgyna* in Skukuza, South Africa (−24.995° lat, 31.595° long) on the 3rd of March, 2023. We photographed specimens of both social parasites ( *T. microgyna* , approx. 10 gynes observed) and hosts ( *T. sericeiventre* ) in situ, participating in nest excavations, using a DSLR camera (Nikon d500 with a 105 mm macro lens). We then hand collected six *T. microgyna* and approximately five *T. sericeiventre* specimens at the nest entrance and put them in 70% ethanol. The total number of gynes is unknown as we could not excavate the whole colony during this chance encounter. We then mounted and imaged a specimen of each species using a Leica DMC5400 camera mounted on a Leica M205C dissecting microscope. We took image montages using the Leica Application suite v. 4.13 and stacked them in Zerene stacker version 1.04. We deposited voucher specimens in the ant collection at the Commonwealth Scientific and Industrial Research Organization in Darwin (Australia) and at the Skukuza Biological reference collection (South Africa).

### 2.1 Dissection

We dissected three *T. microgyna* gynes (two with 2 or 4 wings, and one without wings) approximately 6 months after they were collected from a single *T. sericeiventre* nest and stored in 70% ethanol. Dissections revealed mating status as assessed by the presence or absence of sperm in the spermatheca. We also assessed body condition including the number and condition of ovarioles to estimate the reproductive potential of *T. microgyna* gynes. Lastly, we quantified the abundance of fat body in the gaster, the nature of visible defensive glands, and the contents of the crop, midgut, and hindgut, as this may relate to a gyne's ability to disperse and invade a host colony successfully.

Gynes were dissected under deionized water. A minutin pin was used to secure the mesosoma to a wax dish, and each tergite of the gaster was removed by slipping superfine watchmaker's forceps beneath the dorsal midline, then gripping and peeling away the cuticle (see Figure S1). The location of globular fat body and its approximate depth (number of layers) was recorded. A stream of water was then used to remove fat that occluded visibility of other organs. The presence of liquid in the crop was noted, along with the number of Malpighian tubules and the presence and character of particulate matter in the midgut and hindgut.

The number of ovarioles was counted in situ under a stereoscope, and again after removal of the entire reproductive tract for slide mounting. The condition of ovarioles and the presence or absence of vitellogenic follicles with developing ova were recorded for each gyne. Unripe ovarioles were thin and strand‐like, with a uniform width across their length without girdling and without translucent or opaque ova or evidence of yellow bodies. Ripe ovarioles were those that contained translucent or opaque developing ova and were plump with a beaded appearance that widened towards the calyx. Before removing the reproductive organs, we also noted the size and condition of glands associated with the sting apparatus.

To determine if *T. microgyna* gynes that performed nest excavation tasks had mated, we made wet‐mount slide preparations of each gyne's entire reproductive tract. We ruptured and smeared spermathecae on the glass slide then examined the slides under a compound microscope (Nikon) at 400× magnification to look for evidence of stored sperm. Each spermatheca was small but clearly defined by a characteristic, recurved tubule. If no sperm were visible, we made smears of the entire reproductive tract posterior to the lateral oviducts and re‐examined the slide. To confirm the feasibility of visualizing sperm in specimens stored in 70% alcohol (rather than fresh frozen without preservative), we first practiced dissections using wet mounts of mated *Solenopsis invicta* (BUREN, 1972) and *Pogonomyrmex badius* (LATREILLE, 1802) foundresses that were either frozen at −20°C without liquid or placed in 70% ethanol for 21–40 days. Sperm were visible in the spermatheca of both test species, despite the potential for wash out or clearing expected during alcohol preservation.

## 3 Results

### 3.1 Behavioral Observations

**FIGURE 5:**
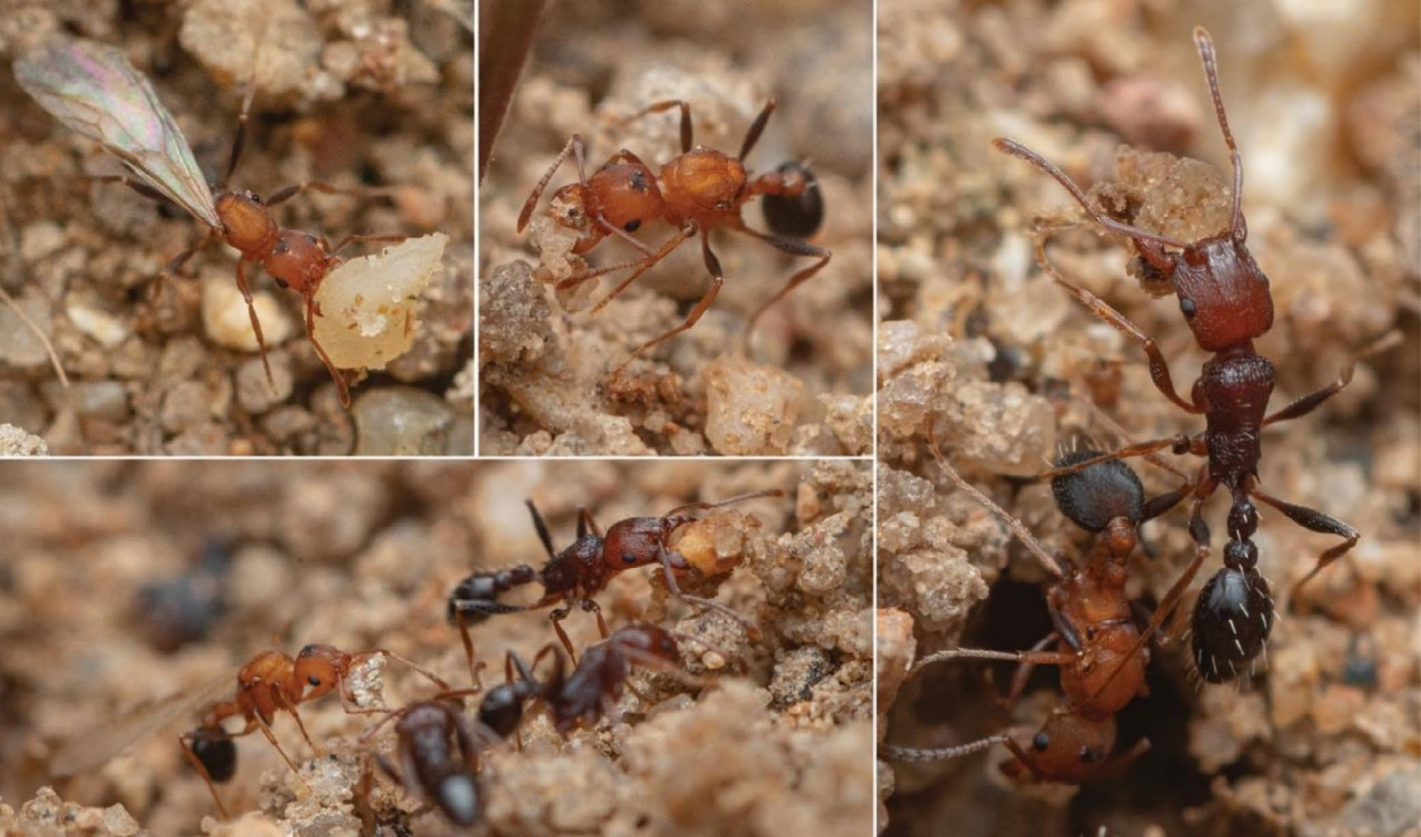
*Tetramorium microgyna* gyne and *T. sericeiventre* workers cooperating on nest maintenance by carrying pebbles and soil within the nest to then deposit them at the periphery of the nest entrance. Note that *T. microgyna* gynes are smaller than their host workers *T. sericeiventre* . Photos by François Brassard.

**FIGURE 6:**
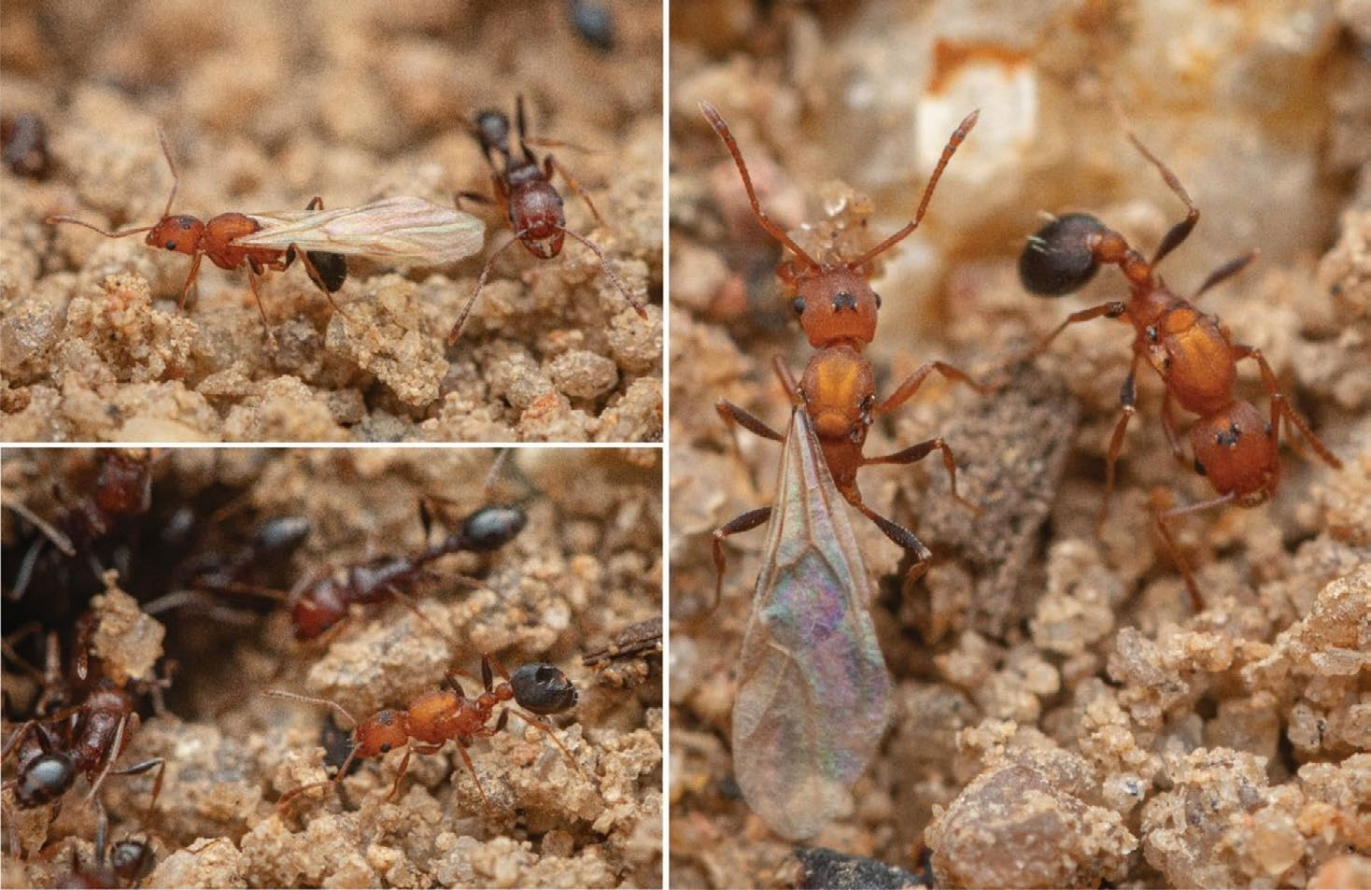
Gynes of *Tetramorium microgyna* at the nest entrance were either winged, wingless, or partially dealate. Photos by François Brassard.

The focal nest of *T. sericeiventre* was in the soil, and host workers were performing nest maintenance and excavation behaviors at the time of our observations. We observed parasitic *T. microgyna* gynes and *T. sericeiventre* workers carrying pebbles and soil from within the nest to then deposit them at the periphery of the nest entrance (Figure 5). Parasitic alate gynes, dealate gynes, and partially dealate gynes all appeared at the nest entrance (Figure 6). All traveled in and out of the nest engaged in repeated substrate deposition behavior.

### 3.2 Dissection

None of the three *T. microgyna* gynes dissected had evidence of sperm in the spermatheca (for consolidated dissection details, see Table S1). Gynes had 6–7 ovarioles (3–4 per ovary). Between 1 and 4 ovarioles were ripe, with forming eggs, while the other ovarioles were inactive and lacked yellow bodies that would suggest any eggs had recently been laid. Gynes had little to no fat body in the gaster, but were well‐fed, as evidenced by the presence of clear liquid in the crop (2 of 3), fine particulate matter that filled the large round midgut, and dense consolidated material in the hindgut. Gynes had fewer than 15 Malpighian tubules, and a large, turgid venom sac/gland that covered the lower 5th of the internal gaster space and attached to the robust sting apparatus, alongside what we presume to be the Dufour's gland (colorless). Large air sacs that flanked the crop were undamaged, inflated, and pearlescent, suggesting that they could have provided the necessary oxygen for powered flight. All dissected gynes were free of internal parasites.

## 4 Discussion

Despite lacking a worker caste, parasitic *T. microgyna* gynes retain the ability to perform a repertoire of non‐reproductive behaviors usually associated with workers. To our knowledge, *T. microgyna* represents the first case of a workerless, inquiline performing nest excavation behavior in a host colony. Worker‐like behavior in unmated gynes with wings (alates) is also exceptionally rare (Fresneau and Dupuy 1988; Table 1), as wing removal can trigger physiological changes associated with worker‐like behavior (Nehring et al. 2012; Pyenson et al. 2022). Assuming that *T. microgyna* is a distinct species from its host based on morphology, several pieces of evidence suggest that gynes involved in nest excavation were failed dispersers, born in the focal nest: (1) The observed parasite gynes were likely unmated, as no sperm was present in the spermathecae of any gyne, (2) mated ant queens typically do not perform nest excavation behavior in the presence of workers, and (3) we observed multiple, non‐physogastric, winged gynes associated with the same focal nest. Although some ant colonies may be parasitized by multiple, reproducing parasite queens, these queens typically do not retain their wings and would have sperm in the spermatheca (Emery 1909; Johnson 1994).

**FIGURE 7:**
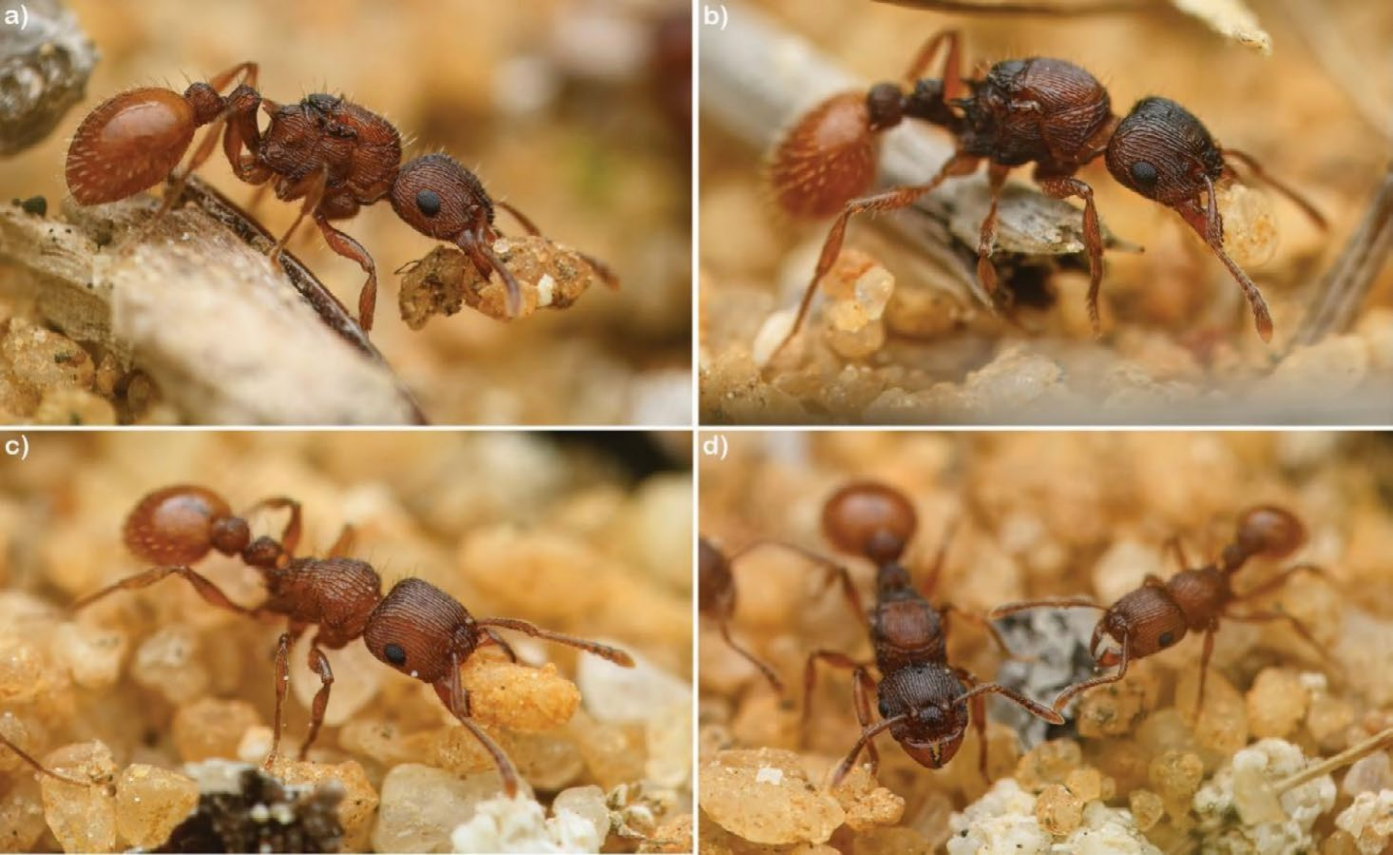
Photos of (a, b) dealate gynes, (c) worker and (d) worker and dealate gyne from a *Tetramorium* sp. (*impressum* gp.). Several dealate gynes (approx. 10) and workers were doing nest excavation, going back and forth from the same entrance of an established nest. No males were seen at the nest entrance, suggesting this was not a nuptial flight. Note in (d) that gynes are larger than workers. Photos taken in Perth (Australia) in 2025 by François Brassard.

Together, our findings suggest that nest excavation by inquiline gynes likely persists due to relaxed selection on post‐reproductive behaviors that were present in a non‐parasitic ancestor. Supporting this is our recent finding that dealate gynes from an established colony of a non‐parasitic *Tetramorium* species exhibit nest excavation behavior (Table 1, Figure 7). Due to their low abundance, the contributions of *T. microgyna* gynes to nest excavation are likely minimal, and unlikely to increase the fitness of the host colony or of parasite kin within the same host nest. Although helping‐behavior in *T. microgyna* might appear to benefit the host colony, the contributions of parasitic gynes could only be considered mutualistic if the fitness benefits of these behaviors outweighed the costs imposed by rearing the parasitic gynes.

Whether nest‐excavation by post‐reproductive inquilines echoes ancestral worker‐like or queen‐like behavior is worthy of discussion. Worker‐like behaviors by unmated gynes, such as foraging, allogrooming, brood‐care, and nest defense, have been reported in several non‐parasitic ant species, representing multiple ant subfamilies (Table 1) (Peeters 1997; Johnson et al. 2007, 2022; Nehring et al. 2012; Vieira et al. 2012; Johnson 2021). Independent foundresses also perform all of the necessary functions of an ant colony, including nest excavation, brood care, and foraging (when not fully claustral) following dispersal, and in the absence of a work force. Likewise, in pleometrotic foundress associations, a division of labor among several foundresses often leads one individual to take on a larger proportion of nest maintenance behaviors, which ultimately increase group survival and fitness of the queen(s) that inherit the nest and its workers (Fewell and Page 1999; Cahan and Fewell 2004; Ostwald et al. 2021). In the case of microgynes, there are currently no reports of newly mated individuals performing social or colony‐founding behaviors such as excavation and brood care. Newly mated microgynes may either integrate back into their own nests after mating (Lenoir et al. 2011) or mate and disperse (Lachaud et al. 1999). We argue that nest excavation by *T. microgyna* is more likely to represent a worker‐like than queen‐like founding behavior because it occurred in the presence of workers in an established host nest. Furthermore, the observed host nest entrance was the approximate width of one worker body length, suggesting that the nest entrance was not being widened in anticipation of a mating flight of the host or parasite species.

### 4.1 Lifetime Reproductive Capacity and Body Condition of Inquiline Gynes

Across ant subfamilies, the number of sperm that ant queens store increases as a function of ovariole number (Tschinkel 1987). *Tetramorium microgyna* gynes have just six ovarioles, which is exceptionally low for the subfamily Myrmicinae (some myrmicines have > 200 ovarioles). Using Tschinkel's (1987) equation, *T. microgyna* gynes are estimated to store just 2000 sperm cells per ovariole, for a total of 12,000 sperm cells. Ants are conservative with sperm use, expending approximately 3.2 sperm cells per fertilized egg (Tschinkel 1987; Tschinkel and Porter 1988). Given *T. microgyna* ovariole number, and assuming 3.2 sperm are spent per fertilization, gynes are estimated to have the capacity to produce ~3700 fertilized eggs per lifetime, and an unknown number of unfertilized, haploid, male eggs. This estimate provides additional evidence that parasitic *T. microgyna* lack the capacity to produce a large number of offspring, or a workforce. This contrasts with species that produce both macrogynes and microgynes, where the number and length of ovarioles is on average lower in microgynes, but there may be significant overlap—suggesting both morphs have the capacity to produce numerous workers (Lachaud et al. 1999).

Dissected gynes had little to no accumulated fat body, but were well fed, as evidenced by the presence of liquid in the crop and particulate matter in the midgut and hindgut. In contrast, independently founding ant queens are typically endowed with large fat reserves amassed during the weeks preceding nuptial flights due to post‐eclosion feeding (Helms and Kaspari 2015). Up to 61% of foundresses' body weight is made up of fat (Keller and Passera 1989), with fat stores located in the mesosoma and gaster (Boomsma and Isaaks 1985). Semi‐claustral independent foundresses, dependent foundresses, and parasitic foundresses are expected to have much less fat than fully claustral, independent foundresses that provision their first larvae from their own fat reserves. For instance, in an interspecific context, semi‐claustral species have on average 18% less body fat than claustral foundresses, whereas dependent foundresses have on average 47% less body fat than claustral foundresses (Keller and Passera 1989).

It is difficult to determine if the *T. microgyna* gynes in our study had a typical amount of fat for parasitic founding, or if they were in such poor condition that they would have been unable to produce more than a few eggs. We can gain some insight from species that employ both independent and auto‐parasitic founding strategies. The red imported fire ant, *Solenopsis invicta*, produces a combination of fat, independently founding queens and lean, dependently founding queens on a seasonal schedule. Dependent foundresses parasitize orphaned conspecific colonies and have 5%–15% less body fat than conspecific independent foundresses produced earlier in the year (Tschinkel 1996). At least one study reports on the fat content of dependent foundresses of *Formica rufa* , which participate in either colony budding or temporary social parasitism of *Formica fusca* . In *F. rufa* , both alate and dealate gynes have almost no fat in their gaster prior to founding, and ovary activation only begins following the histolysis of wing muscles after dispersal (Fedoseeva and Grevtsova 2020).

All 3 *T. microgyna* gynes dissected in our study had at least one active ovariole, with 1–4 eggs forming. It is possible that these eggs were produced in anticipation of mating, and less probable that they were trophic eggs (given that inquilines do not feed their own young). Some ant queens do begin to produce eggs prior to dispersing, and developing oocytes have been found in unmated, dealate gynes (Vieira et al. 2012) including parasitic foundresses of the fire ant, *Solenopsis invicta* (Helms 2018). This contrasts with independent foundresses of *S. invicta* , which develop ovaries only after dispersing (Helms 2018). Although we do not know if *T. microgyna* gynes mate before dispersing, or if they use their wings to disperse, 2 of the 3 parasitic gynes observed performing nest maintenance behavior were partially or fully dealate, but with an intact tracheal system and no other internal anomalies that would prevent powered flight. If the premature loss of wings alone prevented participation in mating or dispersal flights, then this reinforces our hypothesis that these gynes failed to mate and disperse, and subsequently assumed worker‐like roles. Describing the mating behavior and dispersal mode of *T. microgyna* will help resolve this question, as it is possible that queens disperse on foot, without the aid of their wings. Although no males were observed, the mating season of *T. microgyna* in South Africa may be in March and April, as the gynes in the current study were observed in March and the *T. microgyna* specimen collected by Bolton in South Africa was collected in April (AntWeb 2025). However, it is possible that the mating season of *T. microgyna* could be much longer, as we found two records (on the *iNaturalist* platform) of the species in South Africa for October, as well as another for April (Table S2). Meanwhile, we found records of alates of their host species for most months of the year (Table S2), which suggests they may have mating flights throughout the year.

### 4.2 Social Parasitism in *T. microgyna*

The gynes of *T. microgyna* have several morphological attributes that are typical of inquilines: they are smaller than their host workers, whereas queens of their hosts are always larger than the workers, and their cuticle is paler and more delicately sculptured than the cuticle of their host workers (Bolton 1980, Figures 3 and 4). However, other *Tetramorium* inquilines have much more derived morphologies, with mostly edentate mandibles, a lack of propodeal spines, extremely small sizes, physogastric queens, and pupoid males (Bolton 1980; Francoeur and Pilon 2011; Wagner et al. 2018, 2021; Vankerkhoven and Dekoninck 2022). In species with more extreme parasitic traits, edentate mandibles and small body size could physically prevent gynes from efficiently performing tasks that require gripping, like nest excavation or host brood care. Given that *T. microgyna* has relatively well‐developed mandibles, the observed excavation behaviors may be unique among inquilines from the same clade.

**FIGURE 8:**
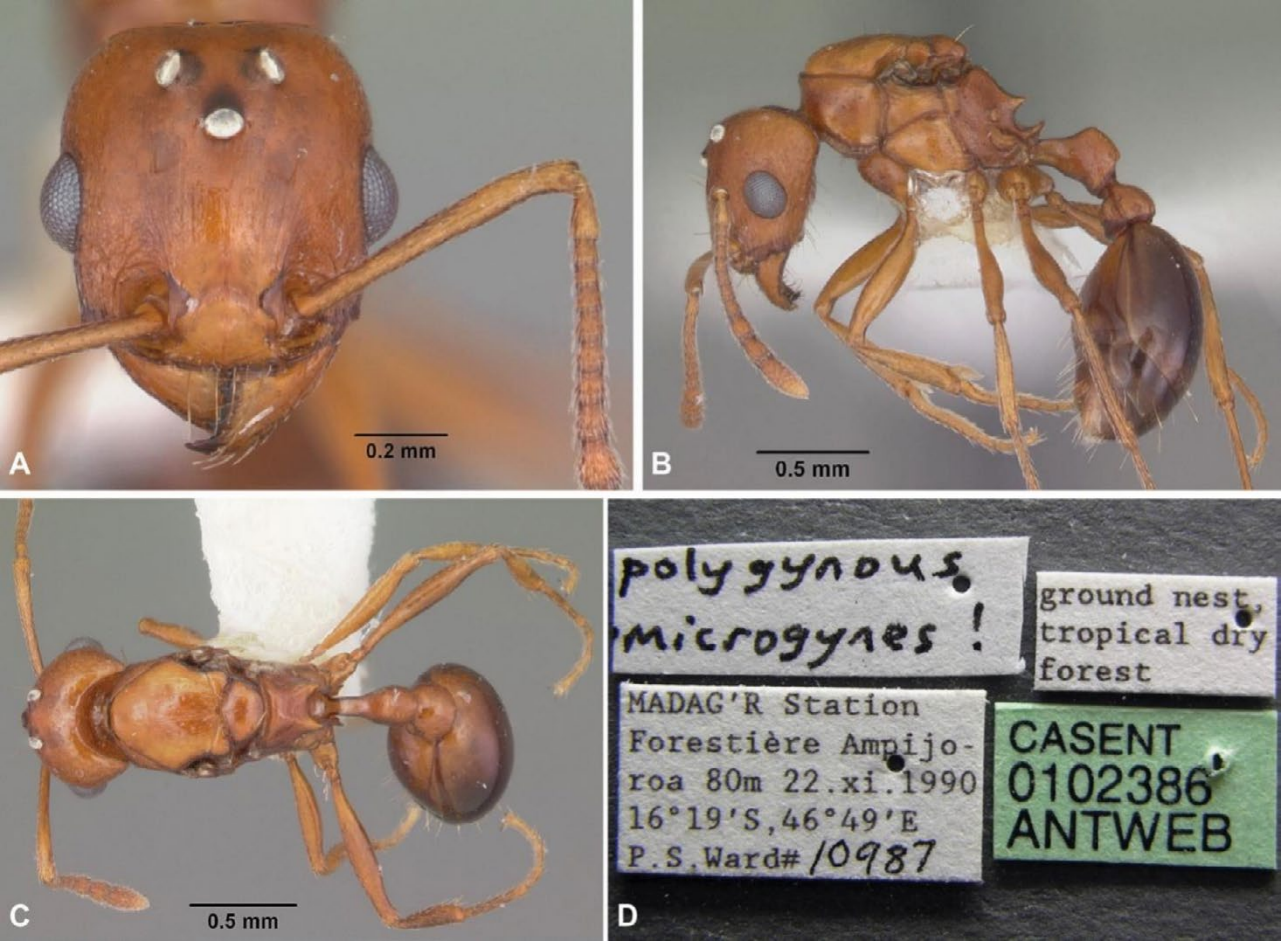
Photos of (A) head view, (B) lateral view, (C) dorsal view and (D) labels of specimen (CASENT 0102386) collected by Philip S. Ward in 1990 in Madagascar. It was then labeled as a microgyne of *Tetramorium quadrispinosum* (now *Tetramorium sericeiventre* ). Photos by April Nobile and taken from AntWeb.org.

**FIGURE 9:**
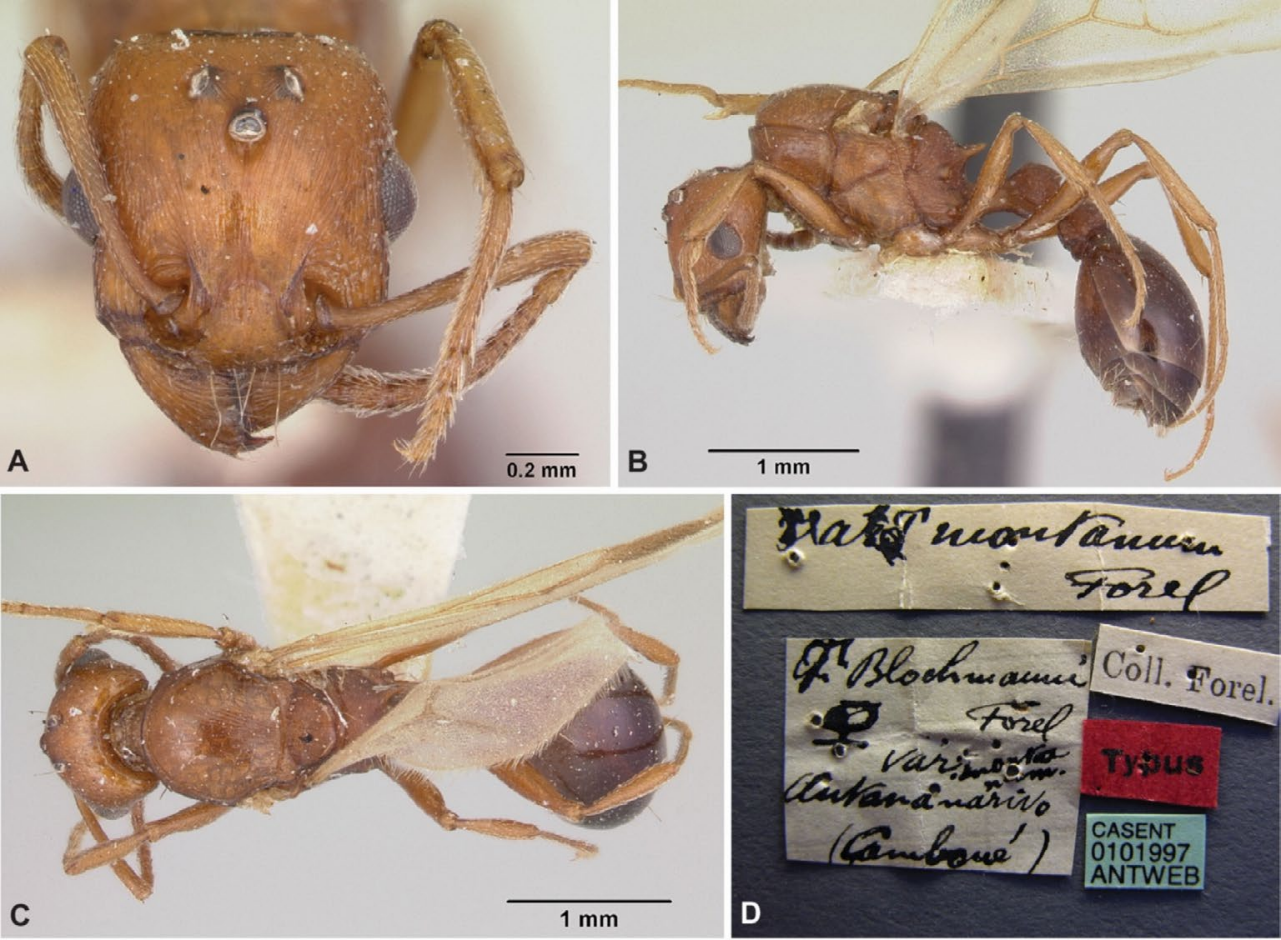
Photos of (A) head view, (B) lateral view, (C) dorsal view and (D) labels of specimen (CASENT 0101997), a typical queen of *Tetramorium sericeiventre* . Photos by April Nobile and taken from AntWeb.org.

So far, we have assumed that *T. microgyna* and *T. sericeiventre* are distinct species. It is possible that putative *T. microgyna* gynes are actually *T. sericeiventre* microgynes (i.e., an alternative, miniature queen morph). This possibility provides an intriguing alternative explanation for the worker‐like behavior observed in *T. microgyna*. In 1990, Professor Philip S. Ward collected in Madagascar what he called microgynes of *Tetramorium quadrispinosum* (now synonymized with *T. sericeiventre* , see Garcia and Fisher 2012). This specimen differs from *T. microgyna* but shows similar morphological features associated with social parasitism, such as a small size, a pale and less sculptured cuticle, and small propodeal spines (Figure 8), especially compared to “typical” queens of *T. sericeiventre* (Figure 9). Whether the specimen represents a separate parasitic species or a conspecific microgyne remains untested. Microgynes have been described as independent social parasite species in the past, only to later be synonymized with their putative host species, although this has led to disagreements, such as in *Myrmica rubra* and *Myrmica microrubra* (Seifert 1994; Steiner et al. 2006; Vepsäläinen et al. 2009). In one case, the putative social parasite *Manica parasitica* was found to be nothing more than a cestode worm infected morph of its presumed host, *Manica bradleyi* (Prebus et al. 2023). We believe it is unlikely that *T. microgyna* is a microgyne, as *T. microgyna* is found within nests of two different host species ( *T. sericeiventre* and *T. sepositum* ), has an extremely low number of ovarioles, and has a derived morphology consistent with social parasitism. Nevertheless, we hope that future molecular phylogenetic analyses of *T. microgyna* , typical *T. sericeiventre* , and *T. sericeiventre* microgynes will one day clarify the species‐level relationships between these organisms.

## 5 Conclusion

In ants, social parasitism is known from 6 subfamilies, 42 genera and 401 species. It occurs predominantly in the diverse subfamilies Dolichoderinae, Formicinae and Myrmicinae (Rabeling 2021), but is also found in relatively species‐poor lineages such as Myrmeciinae (Mera‐Rodríguez et al. 2023). Social parasitism in ants evolved independently at least 91 times across the world (Gray and Rabeling 2023). Despite the rich diversity of socially parasitic ant species, our knowledge of the intricacies of social parasite behaviors mostly comes from a few well‐studied species of Northern latitudes belonging to the genera *Formica*, *Polyergus*, *Temnothorax* and *Harpagoxenus* (Creighton 1927; Talbot 1967; Talbot 1976; Cool‐Kwait and Topoff 1984; Heinze et al. 1994; Foitzik et al. 2001; Bauer et al. 2009; Chernenko et al. 2013 but see Sumner et al. 2003). This geographic focus on social parasitism may result from its increased frequency at higher latitudes (i.e., it follows an inverse latitudinal diversity gradient), especially in the Northern hemisphere (Gray and Rabeling 2023). For instance, up to 30% of the Swiss ant diversity consists of social parasites, compared to roughly 2% of the world's species (Kutter 1968). Nevertheless, social parasitism does occur in the southern hemisphere, albeit in relatively fewer taxa. Most social parasite species are rare and remain poorly known, with most species lacking natural history observations (Rabeling 2021; Gray and Rabeling 2023). As such, there is likely a range of novel behaviors to record in social parasites, including those described in the current study.

As a clade, *Tetramorium* is fascinating because it contains species that span the whole spectrum of the social parasite syndrome. Most social parasite species are extremely rare, but *Tetramorium* is a diverse and ubiquitous genus throughout the Old World in need of phylogenetic treatment. As such, it is likely that many more socially parasitic species are currently unknown. Moreover, our knowledge of the behavior and life history of described social parasites is patchy at best. For example, nothing is known about the host deception, mate choice, mode of dispersal, sex ratio, and abundance of *T. microgyna*. Gaining more information on social parasitism in *Tetramorium* would better our understanding of the evolution and ecology of ants as a whole. As such, we encourage fellow myrmecologists to prospect for such species, and in addition to reporting on morphology and molecules, record the behavior of social parasites.

## Author Contributions

**François Brassard:** conceptualization (equal), investigation (equal), methodology (equal), visualization (lead), writing – original draft (lead), writing – review and editing (equal). **Christina Kwapich:** conceptualization (equal), investigation (equal), methodology (equal), supervision (lead), writing – original draft (supporting), writing – review and editing (equal).

## Funding

This work was supported by Holsworth Wildlife Research Endowment, University of Central Florida, Charles Darwin University, Forrest Research Foundation.

## Conflicts of Interest

The authors declare no conflicts of interest.

## Supporting information


**Data S1:** Supporting Information.

## Data Availability

All data used for this study are available within the main manuscript and Supporting Information.
