# Correction to “Novel Worker‐Like Behavior Observed in Gynes of the Social Parasite *Tetramorium microgyna*”

**DOI:** 10.1002/ece3.73153

**Published:** 2026-02-17

Brassard F. and C. Kwapich. 2026 “Novel Worker‐Like Behavior Observed in Gynes of the Social Parasite *Tetramorium microgyna*.” *Ecology and Evolution* 16 no. 1 e72960. https://doi.org/10.1002/ece3.72960.

The author would like to include the following statement at the end of the acknowledgments section to express gratitude to a collaborator who discovered and photographed the specimens shown in Figure 7:

“We thank Marco Chan for finding the nest of the Australian *Tetramorium* specimens featured in Figure 7.”

We apologize for this error.

