# Supplementary material for: Novel Worker‐Like Behavior Observed in Gynes of the Social Parasite Tetramorium microgyna
Source: Ecol Evol. 2026 Jan 22;16(1):e72960. doi: 10.1002/ece3.72960 (PMC12826340; doi:10.1002/ece3.72960)
Supplement: Supplementary file 1 — Data S1: Supporting Information. [file ECE3-16-e72960-s001.docx]

**Novel worker-like behaviour observed in gynes of the social parasite *Tetramorium microgyna***

François Brassard^1,2*^, Christina Kwapich^3^

^1^Charles Darwin University, Ellengowan Dr, Casuarina, Darwin NT 0810, Australia

^2^School of Agriculture and Environment, The University of Western Australia, Crawley, WA 6009, Australia

*Corresponding author: [francois.brassard.bio@gmail.com](mailto:francois.brassard.bio@gmail.com)

^3^University of Central Florida, Department of Biology, Orlando, Florida, U.S.A.

**Appendix**

**Table S1** Dissection details for three gynes of *Tetramorium microgyna*.

| **Gyne ID** | **Wings** | **Sperm in spermatheca?** | **Number of ovarioles** | **Number of ripe ovarioles** | **Fat body** | **Air sacs** | **Crop** | **Midgut** | **Malpighian tubules** | **Hindgut** | **Poison gland/sac** | **Dufour's gland** |
| --- | --- | --- | --- | --- | --- | --- | --- | --- | --- | --- | --- | --- |
| Tmicro01 | Both wings present on right side of body, wings absent on left side | No | 6 (3 left, 3 right) | 3 moderately ripe (two left ovary, 1 right ovary) | none, completely devoid of fat body | inflated | Full, clear liquid | Full, with fine, dark particulate matter | <15 | Full, single large oblong pellet. Very hard and comprised of small, compressed tubules of particulate matter | Present, large and turgid | visible |
| Tmicro02 | Dealate, fresh wing scars with frayed wing membrane | No | 6 (3 left, 3 right) | 4 moderately ripe (three left ovary, 1 right ovary) | 1-layer thick, minimal coverage with some anterior and dorsal to crop, and ventral near distal end of the gaster | inflated | deflated, empty but not perforated | Full and round with pale, particulate matter | <10 | Minimal brown hindgut material, tubular under magnification as in Tmicro01 | Present, large and turgid | visible |
| Tmicro03 | 4 wings, intact | No | 7 (3 left, 4 right) | 1 ripe with well-developed, opaque egg (right ovary) | 1-layer thick, ventral, beneath all organs, no dorsal fat observed | Deflated or perforated during dissection | Full, clear liquid | Full, with fine, dark particulate matter | Not counted | Empty | Present, large and turgid | visible |

**Table S2** Collection of *iNaturalist* records of reproductive castes for species within the *Tetramorium sericeiventre* species group.

| **species** | **caste** | **host** | **day** | **month** | **year** | **country** | **locality** | **lat** | **long** | **inaturalist id level** | **note** | **url** |
| --- | --- | --- | --- | --- | --- | --- | --- | --- | --- | --- | --- | --- |
| *T. microgyna* | dealate gyne | unknown | 22 | 4 | 2020 | South Africa | Riebeeck West | -33.346 | 18.868 | research grade | identified as *T. sericeiventre*, but this appears to be a *T.* *microgyna* | https://www.inaturalist.org/observations/42836672 |
| *T. microgyna* | alate and partially dealate gynes | *T. sepositum* | 9 | 10 | 2021 | South Africa | Krugersdorp | -26.080 | 27.856 | research grade | Among *T. sepositum* workers. Male photo too but unclear which species it is. Misidentified as same species. | https://www.inaturalist.org/observations/97672142 |
| *T. microgyna* | alate gyne | unknown | 14 | 10 | 2022 | South Africa | West Coast | -33.386 | 18.912 | needs id | identified as *T. sericeiventre*-complex, but this appears to be a *T. microgyna* | https://www.inaturalist.org/observations/139623085 |
| *T. sericeiventre* | alate queen and males | NA | 30 | 3 | 2014 | South Africa | Cape town | -34.053 | 18.457 | research grade | individuals dead and found in pool | https://www.inaturalist.org/observations/10946693 |
| *T. sericeiventre* | dealate gyne | NA | 26 | 8 | 2015 | South Africa | West Coast | -32.138 | 19.184 | needs id |  | https://www.inaturalist.org/observations/11080599 |
| *T. sericeiventre* | males | NA | 3 | 11 | 2015 | South Africa | West Coast | -33.147 | 17.993 | research grade | approx. 5-10 individuals | https://www.inaturalist.org/observations/11102861 |
| *T. sericeiventre* | male | NA | 4 | 11 | 2015 | South Africa | West Coast | -33.166 | 18.055 | needs id | individual is dead | https://www.inaturalist.org/observations/11102876 |
| *T. sericeiventre* | dealate gyne | NA | 6 | 2 | 2017 | South Africa | Cape town | -33.888 | 18.514 | research grade | 2 dealate gynes | https://www.inaturalist.org/observations/11223501 |
| *T. sericeiventre* | dealate gyne | NA | 27 | 12 | 2020 | South Africa | Riebeeck West | -33.346 | 18.868 | research grade |  | https://www.inaturalist.org/observations/67135292 |
| *T. sericeiventre* | queen and alate gynes | NA | 25 | 10 | 2023 | South Africa | Cape Winelands | -33.933 | 18.873 | needs id | a dozen alate gynes or more. Seems like they were uncovered in a nest. | https://www.inaturalist.org/observations/191252453 |
| *T. sericeiventre* | queen | NA | 20 | 8 | 2025 | South Africa | Overberg | -34.158 | 19.721 | needs id | appears to be a queen uncovered in a nest | https://www.inaturalist.org/observations/324831655 |
| *T. sericeiventre* complex | dealate gyne | NA | 5 | 12 | 2021 | South Africa | Colchester | -33.695 | 25.818 | needs id |  | https://www.inaturalist.org/observations/133925777 |
| *T. sericeiventre* complex | dealate gyne | NA | 28 | 10 | 2022 | South Africa | West Coast | -33.347 | 18.868 | needs id |  | https://www.inaturalist.org/observations/140236211 |
| *T. sericeiventre* complex | dealate gyne | NA | 26 | 1 | 2023 | South Africa | West Coast | -32.908 | 18.176 | needs id | individual is dead | https://www.inaturalist.org/observations/147526039 |
| *T. sericeiventre* complex | dealate gyne | NA | 2 | 11 | 2023 | South Africa | Dwarskersbos | -32.689 | 18.238 | needs id |  | https://www.inaturalist.org/observations/190484202 |
| *T. sericeiventre* complex | dealate gyne | NA | 27 | 11 | 2023 | South Africa | Riebeeck West | -33.346 | 18.868 | needs id |  | https://www.inaturalist.org/observations/192799181 |
| *T. sericeiventre* complex | dealate gyne | NA | 25 | 5 | 2024 | Uganda | Busiiro | 0.413 | 32.328 | needs id | in container, perhaps found while foraging to found a colony | https://www.inaturalist.org/observations/219148003 |
| *T. sericeiventre* complex | alates and dealate gynes, and males | NA |  | 6 | 2024 | Gambia | Lower Nuimi | 13.526 | -16.50 | needs id | several dozen individuals | https://www.inaturalist.org/observations/223948835 |
| *T. sericeiventre* complex | dealate gynes | NA |  | 7 | 2024 | Gambia | Lower Nuimi | 13.445 | -16.50 | needs id | 3 gynes on wall | https://www.inaturalist.org/observations/228796025 |
| *T. sericeiventre* complex | dealate gyne | NA | 9 | 1 | 2025 | South Africa | Port Elizabeth | -33.692 | 25.76 | needs id | on ground seemingly looking to found a nest. | https://www.inaturalist.org/observations/324831655 |

**
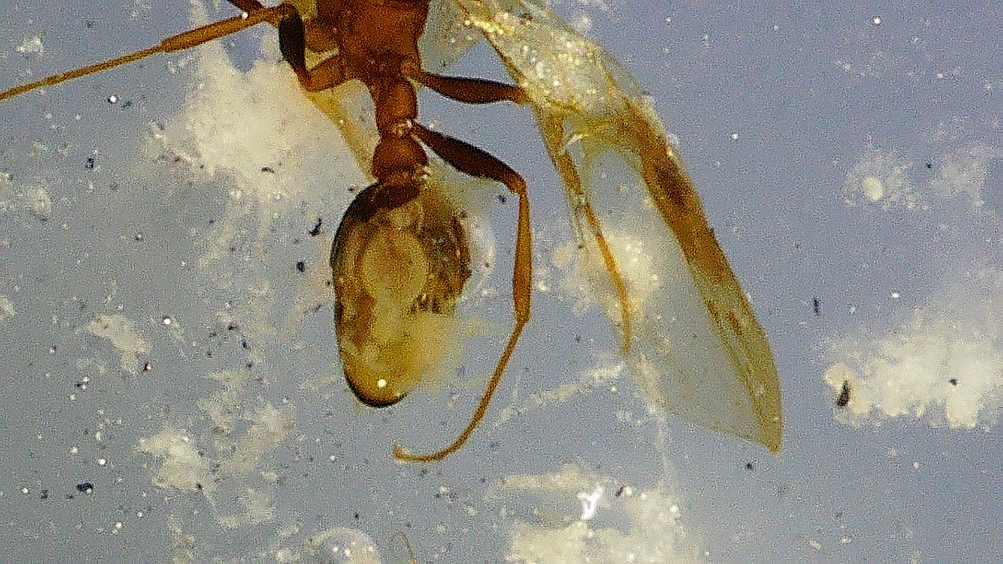

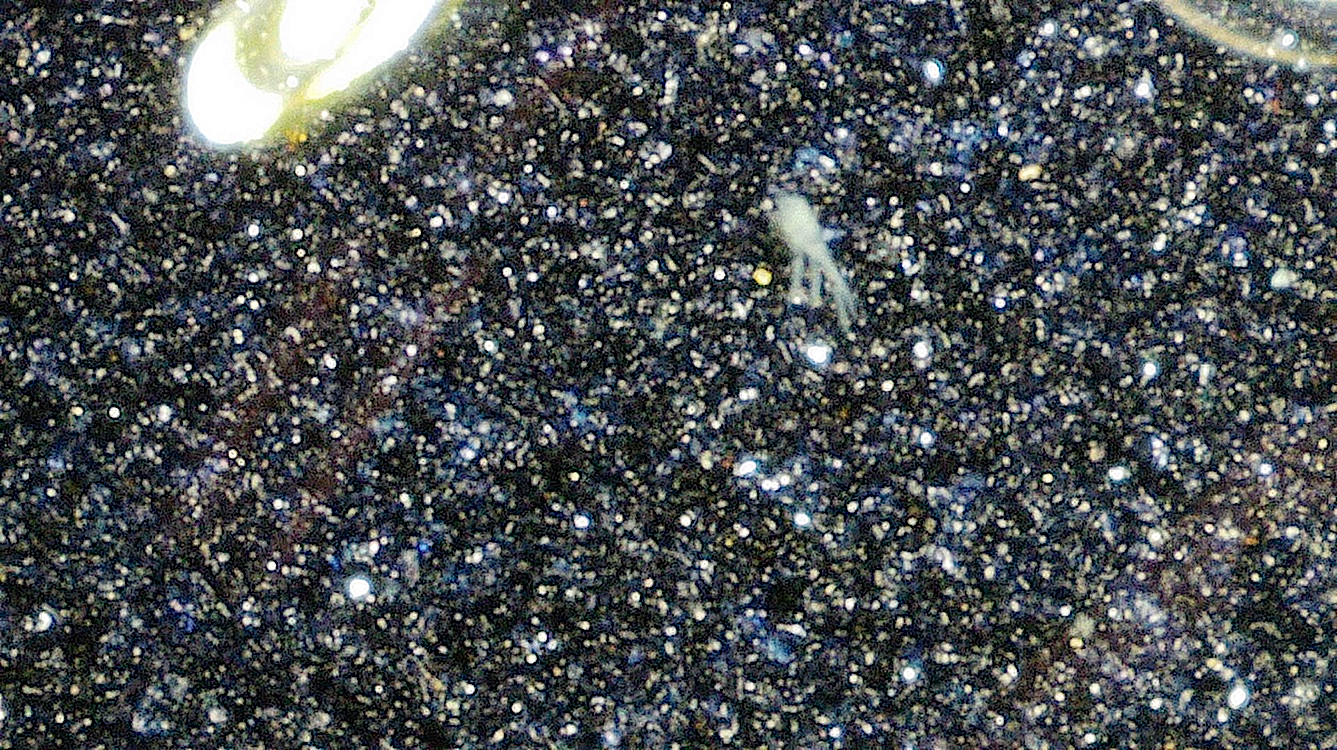

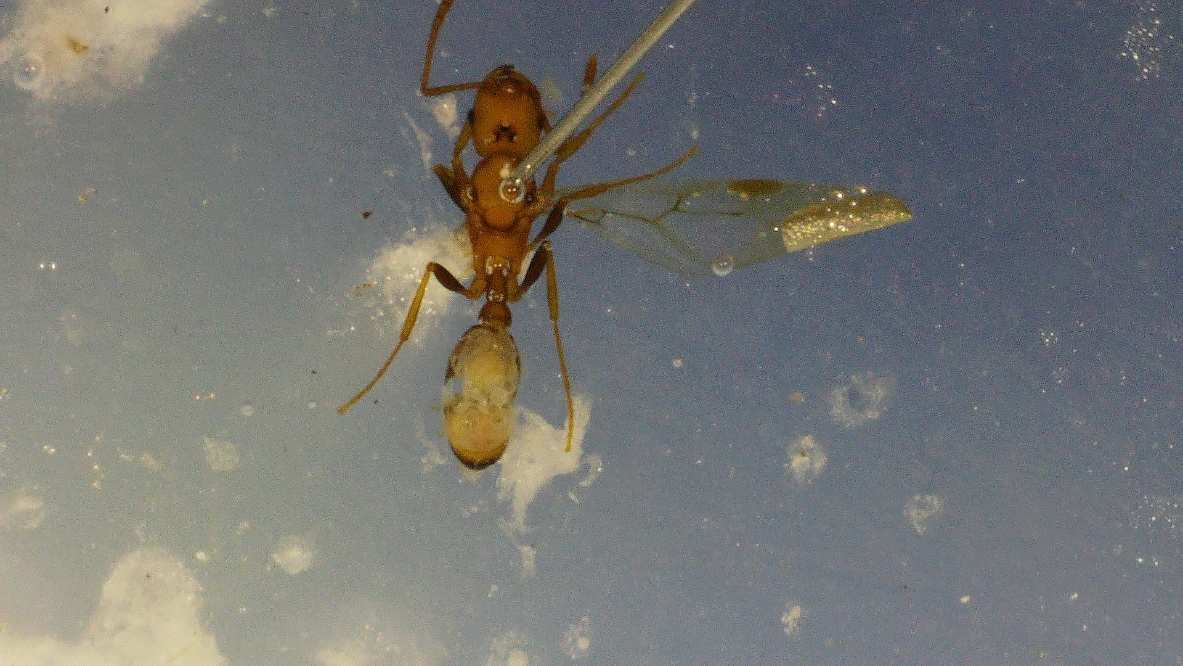
**

**Figure S1.** Internal organs of one *Tetramorium microgyna* gyne (Tmicro01) following removal or tergites, before the dissection of organs (Left and middle). Note the lack of fat body (Left), full gut (middle), and ovarioles with developing oocytes (right).
